# Using social media in Kenya to quantify road safety: an analysis of novel data

**DOI:** 10.1186/s12245-022-00432-6

**Published:** 2022-06-28

**Authors:** J. Austin Lee, Lyndsey Armes, Benjamin W. Wachira

**Affiliations:** 1 Department of Emergency Medicine, Brown University Warren Alpert Medical School, Providence, RI USA; 2 School of Public Health, Brown University, Providence, RI USA; 3 The Aga Khan University, Nairobi, Kenya

**Keywords:** Road safety, Road traffic, Trauma, Kenya, Social media, Twitter

## Abstract

**Background:**

Road traffic injuries are a large and growing cause of morbidity and mortality in low- and middle-income countries, especially in Africa. Systematic data collection for traffic incidents in Kenya is lacking and in many low- and middle-income countries available data sources are disparate or missing altogether. Many Kenyans use social media platforms, including Twitter; many road traffic incidents are publicly reported on the microblog platform. This study is a prospective cohort analysis of all tweets related to road traffic incidents in Kenya over a 24-month period (February 2019 to January 2021).

**Results:**

A substantial number of unique road incidents (3882) from across Kenya were recorded during the 24-month study period. The details available for each incident are widely variable, as reported and posted on Twitter. Particular times of day and days of the week had a higher incidence of reported road traffic incidents. A total of 2043 injuries and 1503 fatalities were recorded.

**Conclusions:**

Twitter and other digital social media platforms can provide a novel source for road traffic incident and injury data in a low- and middle-income country. The data collected allows for the potential identification of local and national trends and provides opportunities to advocate for improved roadways and health systems for the emergent care from road traffic incidents and associated traumatic injuries.

## Background

Road traffic injuries are a growing cause of morbidity and mortality in low- and middle-income countries (LMICs), with a particularly high burden in Africa, including in Kenya [1–3]. In Kenya road traffic incidents are among the leading causes of both morbidity and mortality [4–9]. Road traffic-associated injuries are particularly common among users of motorcycles and public transportation (mutatus/mini-bus), as well as among pedestrians [4, 8, 10]. Broadly, across age groups, Kenyans have poor seatbelt and helmet utilization [11]. Among a cohort of head injured patients in a Kenyan emergency department (ED), none reported seatbelt or helmet use at the time of injury [12]. Furthermore, the Kenyan public has recognized the key role vehicles and over crowded public transit play in increasing risk of injury on Kenyan roadways [13, 14].

Yet, systematic data collection for traffic incidents is broadly lacking across Africa, as well as in Kenya. The ability to target road and traffic safety improvements with tailored solutions requires an understanding of the burden of disease and the current strengths and weaknesses. Robust and accurate statistics around road traffic deaths, hospital data, population surveys, or police reports are often not available [1, 2]. Prior work in Kenya has identified the need to “improve the collection and availability of accurate [road traffic injury] data” in the country [15]. There is recognized underreporting, variance from recognized international standards, and overall inadequate data collection around road traffic incidents in Kenya [16]. Previously, the World Health Organization has estimated that nearly 80% of road traffic fatalities were unreported in prior Kenyan government data [17].

The majority of Kenyans have access to mobile telephones (91% penetration per capita, compared to 80% across Africa) and many Kenyans use social media (an average of nearly 3 hours per day), including an estimated 50% of Kenyans who use the popular microblog and social media platform Twitter [18]. Prior research has evaluated crowdsourced data from mobile phone data and social media platforms such as Twitter to provide timely incident detection [19]. However, the preponderance of prior work in the space has been performed in high-income countries with robust and publicly available crash data, utilized real time GPS or accelerometer data, or had evaluated unique newsworthy events, as opposed to routine social media postings [20–22].

This study aims to evaluate publicly available social media posts regarding road traffic incidents in Kenya. We hypothesize that the data from Twitter can be used as one of the few publicly available sources of road traffic incident data in Kenya given the paucity of available government or other reliable data sources. Furthermore, we detail the epidemiology of Kenyan road traffic incidents using information contained in Twitter posts.

## Methods

### Study design and setting

This was an observation, prospective cohort study, wherein all identified road traffic incidents from February 1, 2019, to January 31, 2021, were included for analysis. Research staff from the

**Fig. 1:**
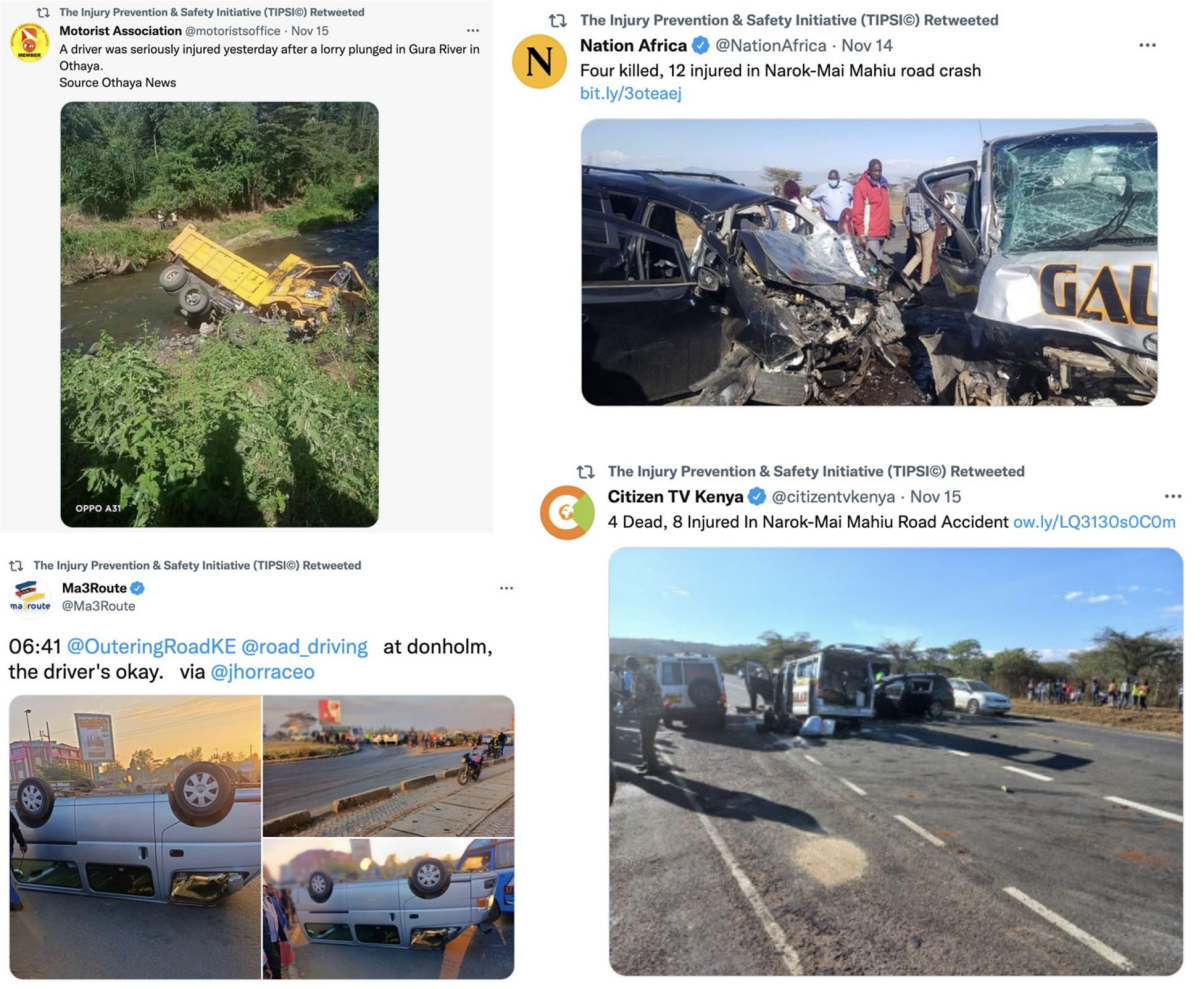
Examples of tweets from @TIPSIKenya

Emergency Medicine Kenya Foundation’s (EMKF) The Injury Prevention and Safety Initiative (TIPSI) actively monitor Twitter daily for reports of road traffic incidents in Kenya (Fig. 1; these examples are from outside of the study period). TIPSI staff then retweet each unique incident onto their Twitter feed (@TIPSIKenya) and take care to ensure no duplicate events are included. Subsequently, research staff abstract key data from each traffic incident from the TIPSI Twitter timeline. Events involving non-motor vehicle incidents, such as boats and planes, were excluded from the analysis.

### Outcomes

Data regarding time of day and extent of injuries were included for analysis when available, based on the details included in a tweet. Information regarding casualties (number involved in an incident), injuries (number reported to have been injured), and fatalities (those who are reported to have died) were captured when available and generally were from what was known or available on scene. Data that is determined some time after an incident, like eventual outcomes such as death for individuals who received hospitalization, was not available and as such not included. Casualties were further divided into whether or not the incident involved vulnerable roadway users (such as motorcyclists, cyclists, and pedestrians).

### Analysis

EMKF TIPSI staff monitor Twitter and abstract data into a Microsoft Excel^©^ database. All statistical analyses were performed using STATA Statistical Software Release 15 (StataCorp LLC, College Station, TX).

## Results

**Fig. 2:**
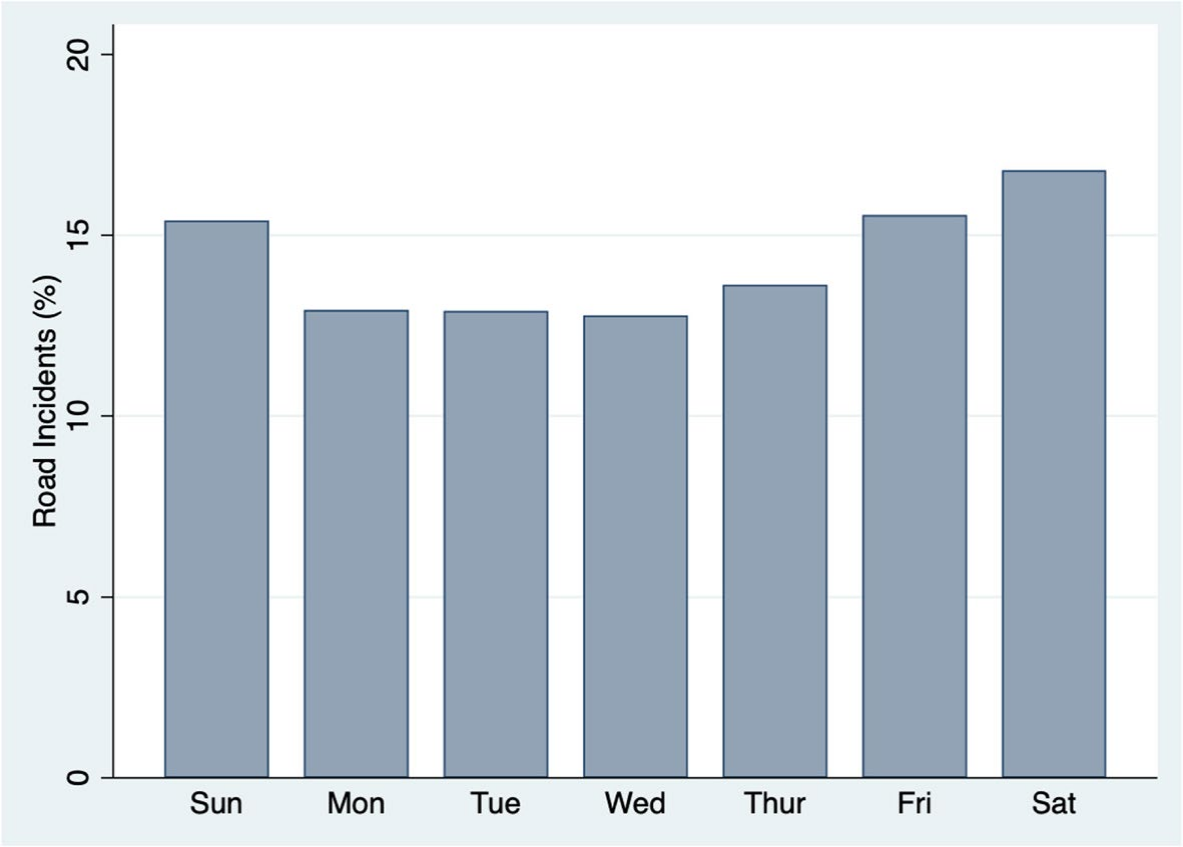
Incidents, by day of the week

**Fig. 3:**
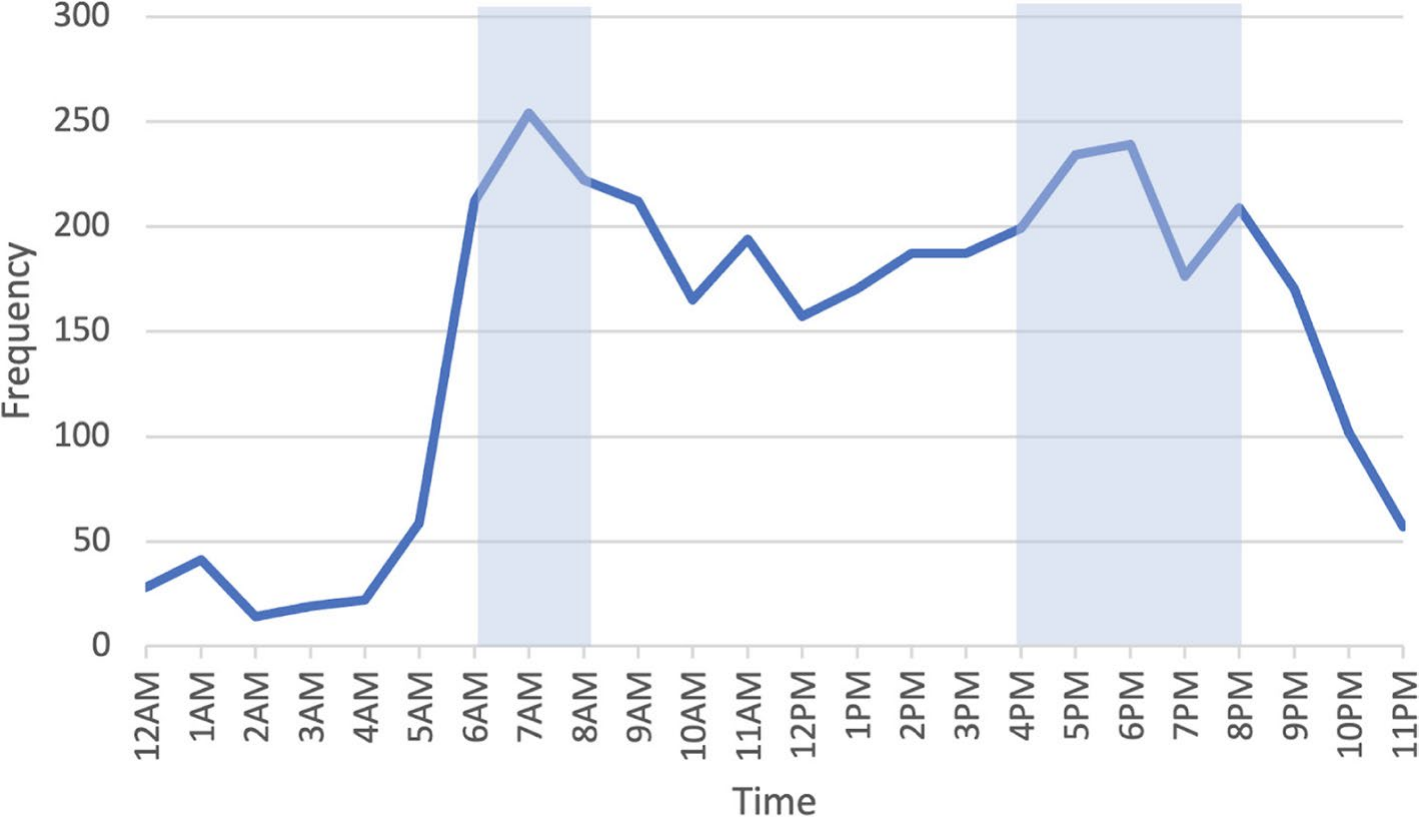
Incidents, by time of day, with highlighted rush hours

The data captured a substantial number of unique road incidents (*n*=3882) from across Kenya during the 24-month study period (Table 1). There was month to month variation in the number of incidents (median monthly incidents: 152), with May 2019 having the maximal incidence (*n*=251, 6.47%). Fridays (15.6%), Saturdays (16.8%), and Sundays (15.4%), account for the highest proportion of incidents (Fig. 2). The majority (*n*=3529, 90.9%) of data have an associated time of occurrence (Fig. 3), and the highest proportion of incidents occurred at 7am (7.2%) and 6pm (6.8%). In particular, rush hour traffic (6:00-8:59 AM, 4:00–8:59 PM) accounts for only 8 hours of the day, but 49.4% (1745/3529) of Twitter-reported road incidents (with an associated time of occurrence) happen during those hours.

**Table 1:** Characteristics of Twitter-reported road traffic incidents in Kenya (February 2019 to January 2021)

**Characteristic**	**Incidents (*n* = 3882)** No. (%)
**Time of day**	
Rush hour (6a–8:59a, 4p–8:59p)	1745 (45.0%)
Non-rush hour	1784 (46.0%)
Missing	353 (9.0%)
**Number of vehicles**	
1	2218 (57.1%)
2	1069 (27.6%)
3 +	137 (3.5%)
Missing	458 (11.8%)

Twitter reports included information about the number of vehicles involved in each incident, revealing that single vehicle incidents were most common (64.47%). There were 1278 (32.9%) tweets that contained information about the casualties from (number of individuals involved in) an incident. Of the tweets reporting types of casualties, 441 (34.51%) involved at least one vulnerable road user. Additionally, a total of 510 tweets (13.1%) reported known human injuries, with a cumulative total of 2043 injuries identified during the data collection period. A further 696 (17.9%) tweets reported at least one fatality, and a cumulative total of 1503 fatalities were recorded.

## Discussion

The methods outlined here allowed for the creation of a dataset with information about unique road incidents in Kenya, a topic of public health interest that has historically lacked sufficient reporting mechanisms. The work by the EMKF TIPSI team to monitor Twitter for unique road traffic incidents in Kenya indicates that this methodology of tracking is a viable source of epidemiologic data in a lower resources setting. It is of particular value given the lack of other publicly available data sources.

Available data from the Kenyan National Transport and Safety Authority indicate higher numbers of fatalities from road traffic incidents than was identified through this dataset [23]. This analysis, with a lower fatality calculation, is somewhat expected given that information gleaned from Twitter presumably only contains information reported from the scene and does not follow up hospital or longer-term morbidity/mortality. Furthermore, though many are, certainly not all road traffic incidents in Kenya are documented by Twitter users.

Previous analysis of a single Kenyan Twitter user (@Ma3route) was focused on geospatial analysis of road traffic incidents and showed significant clustering of incidents at several high risk locations and intersections [24]. This Twitter user is frequently found in the @TIPSIKenya feed. Interestingly, one analysis of a small subset of @Ma3route data showed that the majority of Tweets with geolocation data were found to be geographically accurate when verified in person, though the authors noted that most tweets about road traffic incidents do not have any associated geolocation data [25].

### Limitations

Several limitations are noted. There are challenges that come due to the manual process of monitoring Twitter for information and incidents, as well as a manual data abstraction process, which leave room for the possibility of missed incidents. Additionally, many Kenyans are active users of Twitter, but many more Kenyans do not use the platform at all. Furthermore, the TIPSI team does not have strict incident inclusion/exclusion criteria. Rather, the goal is to catalog any incident and all available data without duplicating previously noted incidents. The initial data source (tweets) are microblogs not meant to be complete or comprehensive public health data. As such, there is no standard format to road traffic data reported on the platform, and may include (or have missing) any number of pieces of information including a location (or reference to a nearby landmark or intersection), the number and types of vehicles, occupants involved, the extent of injuries or fatalities, and may or may not include photographs of the scene. The disparate information is unique to every tweet as the source social-media users provide data input on an ad hoc basis.

### Implications

Road traffic accidents are a major cause of morbidity and mortality in Kenya, and while Twitter can provide some data for context, there is an urgent need to improve the quantification of the road traffic incidents in the country. Improved data would comprehensively include: geolocation, types and number of vehicles, numbers of occupants, types of injuries, the time of occurrence. Such improved data collection will make it possible to better assess the health and economic losses from road traffic incidents, and allow for improved advocacy and a more robust financial argument for improved roadways and road traffic safety. Future work should evaluate the ability for the existing Kenyan emergency medical care systems to adequately treat injuries and longer-term morbidity that results from road traffic incidents.

## Conclusion

Twitter and other digital media platforms can provide a novel source for road traffic incident and injury data. In Kenya, the EMK Foundation TIPSI team has been able to collate data through routine social media monitoring. In Kenya, and in other LMICs, cell phones are pervasive and access to social media platforms such as Twitter provide a data source that can be used for public health epidemiology and advocacy. In countries or settings with inadequate systematic data collection of road traffic incidents, Twitter and other social media platforms may provide the best available data source and can serve as an important tool for advocacy and public health improvement. Furthermore, cell phones and social media platforms are well suited to provide support and enhance timely trauma care. In Kenya, the ability to use a cell phone or social media platform to quickly identify health facilities capable of providing trauma care and orient individuals on how to quickly get to such facilities is of urgent importance.

Twitter can provide a novel method for identification and collation of road traffic incidents in Kenya and other LMICs. The data collected allows for the identification of local and national trends and provides the potential opportunities to advocate for improved roadways and health systems for the emergent care from road traffic incidents and associated traumatic injuries.

## Data Availability

The datasets generated and analyzed during the current study were obtained from and are available at https://twitter.com/TIPSIKenya

## Abbreviations

LMICs: Low- and middle-income countries
ED: Emergency department
EMKF: Emergency Medicine Kenya Foundation’s
TIPSI: The Injury Prevention and Safety Initiative
